# Gout, Rheumatism, and Rheumatoid Arthritis

**Published:** 1900-05-26

**Authors:** 


### Hospital Clinics and Medical Progress.

### GOUT, RHEUMATISM, AND RHEUMATOID ARTHRITIS.

While acute rheumatism and frank inflammatory gout are diseases which it is easy to differentiate, much confusion is often met with in regard to the more chronic cases. Nor is this to be wondered at when we consider how little resemblance there is between chronic rheumatism or chronic gout, and the corresponding acute diseases, that in their chronic forms both these maladies have also to be separated from chronic rheumatoid arthritis, that disorder which, apparently from the fact that it is neither gout nor rheumatism, has been called, and is still called by many, rheumatic gout. It is, however, of the utmost importance that all such cases should be properly classed, for their treatment differs greatly, and success in their management depends largely upon correctness of diagnosis.

Dr. Luff[1] points out that a clinical test in the diagnosis of chronic articular affections, which, although a rough one, is frequently of much assistance, is to ascertain the effect of treatment with salicylate of soda. If the case responds well to this treatment the disease is most probably rheumatism, while if it does not the existence of rheumatoid arthritis or of gout is fairly certain, as neither of these affections responds well to salicylates. Then although rheumatism in its chronic and subacute forms may certainly affect the joints it never leads to that gross permanent deformity which the other affections may produce, especially to the lipping of the cartilages, the bony outgrowths, and the grating of the ends of the bones which are found in rheumatoid arthritis. In the condition known as *rhumatisme fibreux* deformity may be met with—a fusiform enlargement of the small joints due to thickening of the joint capsule—but there is no lipping and no osteophytic outgrowths, so that the separation of chronic rheumatism from the other condition is usually a fairly easy matter. There are pains in the joints, pains which fly about from joint to joint and generally affect some of the muscles at the same time; if in connection with these flying pains there are indications of rheumatic erythema—erythema nodosum—the diagnosis of rheumatism is fairly obvious, and the response of the disease to salicylates settles the diagnosis. Between rheumatoid arthritis and gout mistakes are more easily made, and the matter is somewhat complicated by the fact—for it almost certainly is a fact—that under the head of "rheumatoid arthritis" several very different conditions are included. Still gout, even in its chronic forms, is a very different disease from the group of conditions which is for the present called rheumatoid arthritis. Dr. Luff says: "Rheumatoid arthritis occurs most commonly in females, gout occurs mostly in males. Rheumatoid arthritis occurs most commonly amongst the poor and ill-nourished, and especially under conditions of depressed health, prolonged anxiety, and exposure to damp and sudden alterations of temperature; gout mostly among the well-to-do and well-nourished. Rheumatoid arthritis is a disease which is improved by good dieting; in the case of a gouty person, a spare and plain diet is indicated. The onset of rheumatoid arthritis is insidious; that of gout, sudden and obvious. As regards the commencement of the attack, gout most commonly begins in one of the feet, especially in the great toe joint; rheumatoid arthritis, although ultimately it affects many joints of both hands, nearly always begins in one joint, most commonly selecting one of the joints of the thumb, either the carpo-metacarpal or metacarpo-phalangeal joint, after which it rapidly spreads to the other joints. In the joint affections of rheumatoid arthritis there is no obvious swelling at first, and no marked redness; in the joint affections of gout, at the commencement of acute or subacute cases there is very obvious swelling, marked redness, and a shiny condition of the skin around the affected joint. In rheumatoid arthritis there is very little pain at first. There is some aching in the joint, but the affection starts in a very insidious manner. . . . Gout, however, begins in the most marked manner with severe pain, the patient, as a rule, waking up in the early morning with excruciating pain in the great toe. Therefore, if doubt exists . . . the patient should be questioned as to the commencement of the attack. . . . An important factor in the differential diagnosis of the two diseases is that there is a joint commonly affected in rheumatoid arthritis which is not affected in cases of gout, viz., the tempo-maxillary articulation. . . . Another distinction is this, and it is perhaps the most important, viz., that in connection with rheumatoid arthritis there is a remarkable symmetry in the affection of the smaller joints of the hands. In gout that symmetry is wanting. . . . Lastly, in a case of simple rheumatoid arthritis, sodium biurate is not found in the joints or tissues, whereas in the gouty person sodium biurate exists in the affected joints and also in other tissues."

A word must be said about the etiology of these different conditions, although our knowledge on that subject is as yet of a somewhat speculative nature. Acute rheumatism is probably an infectious or, in other words, a microbic disease, as shown by its occasionally occurring in outbreaks, by the way in which in a certain house a number of the family will be seized with it, and by the suddenness with which people are attacked. As to gout, there is no doubt that this is a disease which is associated with an accumulation in the circulation of uric acid in the form of the sodium quadriurate at first, and afterwards in the form of the biurate, and, whatever differences there may be as to where the uric acid is produced and other matters, there can be no doubt that the deposition of sodium biurate is intimately related to the gouty attack.

In regard to the etiology of rheumatoid arthritis there is much difference of opinion. It is probable that under this name several distinct diseases are included, some of which would seem to be infective diseases, due to micro organisms settling in the joints, and, by the toxins they pour into the circulation, producing the nervous symptoms so commonly met with in connection with this disease.

The manner in which these several diseases are associated is a matter of some interest. Rheumatism certainly predisposes to rheumatoid arthritis by impairing the nutrition of the joints, and leaving them in a condition fit for the development of the micro-organism by which the rheumatoid disease is caused.

Then, again, although it is not common to find rheumatoid arthritis and gout associated in the same individual, this complication does sometimes arise. What happens, according to Dr. Luff, is that if a patient with rheumatoid arthritis indulges in rich living for a lengthened period, and especially if he takes much wine, he may develop gout. Gouty deposits may thus be found in his joints, even in the very joints affected with the arthritis. But this is a mere accident. There is no reason to believe that the one condition predisposes to the other. The point of interest is this—that even while denying the rheumatic or gouty nature of "rheumatic gout," one has to admit that an intermingling of diseases may take place to this extent; that rheumatoid arthritis may occur in a rheumatic joint; and that gout may complicate a rheumatoid arthritis.

[1] Practitioner, May.

